# *HABP2* germline variants are uncommon in familial nonmedullary thyroid cancer

**DOI:** 10.1186/s12881-016-0323-1

**Published:** 2016-08-17

**Authors:** Alexia L. Weeks, Scott G. Wilson, Lynley Ward, Jack Goldblatt, Jennie Hui, John P. Walsh

**Affiliations:** 1 Department of Endocrinology & Diabetes, Sir Charles Gairdner Hospital, Nedlands, WA 6009 Australia; 2 School of Medicine & Pharmacology, The University of Western Australia, Crawley, WA 6009 Australia; 3 Department of Twin Research and Genetic Epidemiology, King’s College London, London, SE1 7EH UK; 4 Genetic Services of Western Australia, King Edward Memorial Hospital, Subiaco, WA 6008 Australia; 5 School of Paediatrics and Child Health, The University of Western Australia, Crawley, WA 6009 Australia; 6 Pathwest Laboratory Medicine WA, Nedlands, WA 6009 Australia; 7 School of Pathology and Laboratory Medicine, University of Western Australia, Crawley, WA 6009 Australia

**Keywords:** Papillary thyroid cancer, Thyroid carcinoma, Oncogenes, *HABP2*

## Abstract

**Background:**

The genetic basis of nonsyndromic familial nonmedullary thyroid cancer (FNMTC) is poorly understood. A recent study identified *HABP2* as a tumor suppressor gene and identified a germline variant (G534E) in an extended FNMTC kindred. The relevance of this to other FNMTC kindreds is uncertain.

**Methods:**

Sanger sequencing was performed on peripheral blood DNA from probands from 37 Australian FNMTC kindreds to detect the G534E variant. Whole exome data from 59 participants from 20 kindreds were examined for mutations in *HABP2* and the thyroid cancer susceptibility genes *SRGAP1*, *NKX2-1, SRRM2* and *FOXE1.* The population prevalence of the G534E variant in *HABP2* was examined in two independent cohorts.

**Results:**

Heterozygosity for the G534E variant in *HABP2* was found in 1 of 37 probands (2.7 %), but did not cosegregate with disease in this kindred, being absent in the proband’s affected sister. From whole exome data, pathogenic mutations were not identified in *HABP2*, *SRGAP1*, *NKX2-1, SRRM2* or *FOXE1.* Heterozygosity for the G534E variant in *HABP2* was present in 7.6 % of Busselton Health Study participants (*N* = 4634, unknown disease status) and 9.3 % of TwinsUK participants (*N* = 1195, no history of thyroid cancer).

**Conclusions:**

The G534E variant in *HABP2* does not account for the familial nature of NMTC in Australian kindreds, and is common in the general population. Further research is required to elucidate the genetic basis of nonsyndromic FNMTC.

## Background

Thyroid cancer is one of the most common endocrine malignancies, accounting for 95 % of malignancies in endocrine organs [1]. The incidence of thyroid cancer is increasing worldwide, and in the US alone has almost tripled in the last 30 years [1, 2]. Nonmedullary thyroid cancers (NMTC) account for over 95 % of thyroid cancers [3]. These cancers are of follicular origin and are comprised of papillary (PTC) (accounting for 85 % of NMTC), follicular (11 %), Hürthle cell (3 %) and anaplastic histotypes (1 %). The remaining 5 % of thyroid cancers, medullary thyroid cancer (MTC), arise from the parafollicular C cells [4].

NMTC shows significant heritability, with an 8 to 10-fold increased risk in first-degree relatives of affected individuals [5]. Nonsyndromic familial nonmedullary thyroid cancer (FNMTC) is thought to account for approximately 3.5–10 % of all NMTC [6]. It is defined by the presence of thyroid cancer of follicular cell origin in 2 or more first-degree relatives, in the absence of a recognized familial cancer syndrome (such as Cowden syndrome or familial adenomatous polyposis) [7]. Both familial and sporadic NMTC are more common in women than men [8].

Whilst it seems clear that there is a hereditary basis to nonsyndromic FNMTC, until recently no specific genetic mutation has been discovered with a confirmed role in etiology [9]. Studies investigating the basis of FNMTC have identified 4 susceptibility genes, *SRGAP1* (SLIT-ROBO Rho GTPase Activating Protein 1), *FOXE1* (Forkhead Box E1), *SRRM2* (Serine/Arginine Repetitive Matrix 2) and *NKX2.1* (NK2 Homeobox 1) [10–12]. A number of susceptibility candidate chromosomal loci were also identified, at 1q21, 6q22, 8p23.1-p22 and 8q24 [13]. It has been suggested that FNMTC is an autosomal dominant condition with incomplete penetrance and variable expressivity, but the exact mode of inheritance remains unknown [14]. Alternatively, FNMTC may be a polygenic disease caused by a low-to-moderate number of low-penetrant alleles [5].

In a recent study, Gara et al. identified *HABP2* (Hyaluronan Binding Protein 2) as a novel causative gene in FNMTC. A single G → A substitution (G534E) in exon 13 was associated with FNMTC in a kindred with 7 affected individuals [15]. Using expression studies and cell transformation assays, the study provided evidence that *HABP2* is a tumor-suppressor gene. The G534E variant was also found in 19 of 423 (4.7 %) multi-ethnic patients with PTC from the Cancer Genome Atlas (TCGA), as compared with 0.7 % of individuals with unknown disease status.

In subsequent editorial correspondence, however, it was reported that the G534E variant in *HABP2* was not detected in 12 FNMTC kindreds or 217 sporadic PTC cases from China [16], and it was suggested that in some populations, the prevalence of the G543E variant was much higher than 0.7 %, and as high as 3–5.7 % [17–19].

Several additional studies have now been published. Tomsic et al. identified the G534E variant in 6.1 % of familial cases, 8.0 % of sporadic cases and 8.7 % of controls, and found that the variant did not cosegregate with NMTC in several kindreds [20]. In a study of 2105 NMTC cases from the British Isles, the frequency of the variant was 4.2 % in cases and 4.6 % in controls [21]. In a study from Saudi Arabia, Alzahrani et al. found no association between the variant and familial or sporadic NMTC compared with controls [22]. These studies, therefore, do not support an association between the variant and NMTC. In contrast, in a study from China, Zhang et al. identified the variant in 6 affected subjects from 4 kindreds, consistent with *HABP2* being a FNMTC susceptibility gene [23].

The association between this variant and NMTC is therefore somewhat controversial. To address this, we investigated a collection of 37 FNMTC kindreds for the presence of this variant using Sanger sequencing. We examined whole exome data from a subset of kindreds for other pathogenic missense variants in *HABP2* and for mutations in *SRGAP1*, *NKX2-1, SRRM2* and *FOXE1*. We also determined the population prevalence of the G543E variant in *HABP2* in two independent cohorts.

## Methods

Starting in November 2009, we recruited affected and unaffected members of kindreds to a study of the genetic basis of nonsyndromic FNMTC, initially using a candidate gene approach and subsequently using whole exome sequencing. The setting of the study was a tertiary referral center in Western Australia. Probands and kindreds were referred by clinicians (endocrinologists, surgeons and nuclear medicine physicians) and also identified from the Familial Cancer Registry of Genetic Services of Western Australia. DNA was extracted from peripheral whole blood using QIAGEN QIAamp DNA Blood mini or midi kits (QIAGEN Pty, Chadstone, Victoria, Australia) according to manufacturer's instructions. Quantification was performed using the Nanodrop2000. Sanger sequencing of exon 13 of *HABP2* (including the G534E variant) was performed on DNA from probands from each kindred using primers as described by Gara et al. [15] Sanger sequencing was performed by the Australian Genome Research Facility (AGRF;www.agrf.org.au) and data analyzed using Geneious (Biomatters Ltd., Auckland, New Zealand). Whole exome capture and sequencing was performed by AGRF and by Lotterywest State Biomedical Facility Genomics (Perth, Western Australia) using Illumina HiSeq2000 and Applied Biosystems 5500 SOLiD systems. Exome capture was performed using *SureSelect* Human All Exon *V5* + UTRs, TargetSeq Exome Enrichment System and TruSeq Exome Enrichment kit. We captured and sequenced coding regions to a mean depth of 68.7× which was sufficient to call variants at ~98 % of each targeted exome. An average of 87,245,482 reads were generated per affected individual as paired end, 100 bp reads. The exome data were annotated using ANNOVAR [24] and examined for pathogenic missense, splice-site, nonsense and start-gained variants in the *HABP2* gene, as well as novel and previously identified variants in the *SRGAP1*, *NKX2-1, SRRM2* and *FOXE1* genes, using Varsifter software [25]. The predictive tools Polyphen-2, SIFT and MutationTaster were used to determine pathogenicity of detected variants and Minor allele frequency data from the 1000 Genomes Project was used to determine population frequency.

The population frequency of the G534E variant was estimated using deeply imputed genome wide association (GWA) data from 4634 participants in the 1994-5 Busselton Health Study (www.bpmri.org.au) a community-based, mainly White cohort from Western Australia. Genotyping was performed using the Illumina 610Q and 660W arrays, with results imputed to the 1000 Genomes (Build 37) reference panel, as previously described [26]. The frequency of the variant by direct genotyping was as determined from next-generation sequence data from 1195 participants in the TwinsUK cohort (www.twinsuk.ac.uk). These participants were euthyroid, with no history of thyroid surgery or thyroxine treatment. Next-generation sequencing was performed using the Illumina HiSeq platform and aligned to GRCh37 human reference sequence as previously described [26].

The study was approved by the Sir Charles Gairdner Group Human Research Ethics Committee (trial no. 2009-128). Written informed consent was provided by all participants.

## Results

We recruited 109 participants from 37 kindreds with nonsyndromic FNMTC to the study. The number of individuals with thyroid cancer ranged from 2 to 6 per kindred; in 28 kindreds there were 2 affected individuals, in 9 kindreds there were 3 or more affected individuals. The ethnicity was Caucasian for 33 kindreds and Asian for 4 kindreds (Table 1).

**Table 1:** Characteristics of 37 FPTC probands

Characteristic	Patients (*N* = 37)
Age at diagnosis (SD)	46.7 (15.9)
Female N (%)	27 (73.0 %)
Ethnicity	
Caucasian	33 (89.2 %)
Asian	4 (10.8 %)
Histological subtype	
Papillary	30 (81.1 %)
Papillary (follicular variant)	6 (16.2 %)
Papillary (tall cell variant)	1 (2.7 %)
Multifocal	25 (67.6 %)
TNM staging^a^	
T1 N0 M0	10
T2 N0 M0	5
T3 N0 M0	1
T4 N0 M0	
T1 N1 M0	5
T2 N1 M0	7
T3 N1 M0	5
T4 N1 M0	3
Staging^a^	
I	23 (63.9 %)
II	3 (8.3 %)
III	6 (16.7 %)
IV	4 (11.1 %)

^a^Available for 36 probands. Detailed histopathology not available for one proband who underwent surgery in 1983

**Fig. 1:**
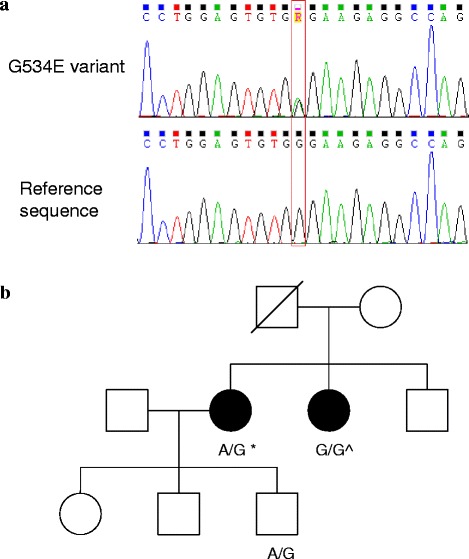
Panel **a** shows the sequence chromatogram of G534E variant in the proband and the reference sequence. Panel **b** shows the pedigree with the *HABP2* genotype for the G534E variant A/G (wild type is G/G). Squares denote male family members, circles female members, shaded symbols affected members and slashes deceased members. *Head and neck irradiation age 16, PTC (T2N1M0) and multiple Hürthle cell adenomas at age 46. ^Multifocal PTC at age 55

Sanger sequencing was performed on peripheral blood DNA from probands from each kindred in order to detect the G534E variant in *HABP2*. Heterozygosity for the G534E variant was detected in a proband from a single kindred (Fig. 1a). The pedigree is illustrated in Fig. 1b. The proband was a white female who underwent facial irradiation for acne at age 16, and presented in 1993 at age 46 with bilateral thyroid nodules. Fine needle aspiration biopsy from the right lobe of thyroid was consistent with Hürthle cell neoplasm, and from the left lobe was consistent with hemorrhage into a thyroid nodule. Total thyroidectomy was performed. Histopathology of the right lobe showed a 30 mm follicular variant of PTC, a 20 mm hemorrhagic Hürthle cell adenoma and two further small foci of PTC. In the left lobe, there were multiple Hürthle cell adenomas up to 25 mm in diameter and a further small focus of PTC. One of 2 excised lymph nodes contained metastatic PTC. Radioactive iodine (3000 MBq) was administered, with no evidence of distant metastases on the post-therapy scan (stage T2N1MO). There has been no evidence of recurrence during follow-up. The proband’s son also has the G534E variant. He has no significant medical history, but has not yet been assessed clinically or sonographically for thyroid neoplasia.

The proband’s affected sister underwent a right hemithyroidectomy in 1985 at age 36 for a thyroid nodule. Histopathology was of follicular adenoma, with an incidental finding of a single focus of papillary microcancer. At age 55 she developed a left sided thyroid nodule and underwent completion thyroidectomy. Histopathology revealed a 13 mm predominantly cystic PTC with an additional 3mm focus of follicular variant of PTC (stage T1NXMX). She did not receive radioiodine treatment and there has been no evidence of recurrence during follow-up. Sanger sequencing on this individual revealed wild type results, without the G534E variant in *HABP2*.

The G534E variant in *HABP2* was not found in probands from the remaining 36 kindreds by Sanger sequencing. Whole exome sequencing data were available from 59 individuals (50 affected, 9 unaffected) from 20 FNMTC kindreds, of which 7 kindreds had 2 affected individuals and 13 kindreds had 3 or more affected individuals, and were examined for the G534E variant and other pathogenic missense variants in *HABP2*; none were identified. The whole exome data were also examined for variants previously reported as associated with NMTC in *SRGAP1* (Q149H, R617C), *FOXE1* (A248G), *SRRM2* (S346F) and *NKX2.1* (A339V) and for novel pathogenic mutations in these genes; none were identified in the kindreds.

The population frequency of the G534E variant was determined from two independent cohorts. Of 4634 participants in the 1994-5 Busselton Health Study (with unknown thyroid cancer status), 351 (7.6 %) were heterozygous for the variant (from imputed genotypes), whereas 4 were homozygous, giving a minor allele frequency of 3.9 %. From directly genotyped next-generation sequence data from 1195 participants in the TwinsUK study who had no history of thyroid cancer or other thyroid diseases, 111 (9.3 %) were heterozygous for the variant and 1 individual was homozygous, giving a minor allele frequency of 4.7 %.

## Discussion

This study examined the frequency of the recently identified G534E variant in *HABP2* in a sample of FNMTC kindreds. We identified the variant in only 1 of 37 kindreds (2.7 %). The proband in this kindred had unusual thyroid histopathology, with multiple Hürthle cell adenomas as well as multifocal PTC. She had previously undergone head and neck irradiation for acne, a well-recognized risk factor for thyroid cancer, which occurs in up to 31 % of individuals receiving this treatment [27]. Her histopathology presumably reflects the extensive mutagenic effects of ionizing radiation combined with genetic predisposition to thyroid neoplasia. In this kindred, the variant did not cosegregate with the disease, being absent in her sibling, who had multifocal PTC. There are two possible explanations for this. The sister may represent a phenocopy, as thyroid cancer can affect two members of the same family by chance or because of shared environmental rather than genetic factors [28]. Alternatively, it may be that these two siblings share pathogenic mutations in as yet unidentified susceptibility genes.

The frequency of the G534E variant carriers in two independent cohorts was 7.6 and 9.3 %, indicating that this is in fact a common polymorphism, rather than a rare variant. The frequency in the 37 probands was somewhat lower (2.7 %), showing that this variant does not explain the familial aggregation of NMTC in the kindreds studied. Gara et al. reported the G534E mutation in 4.7 % of a sample of patients with PTC from TCGA [15], and our results are broadly consistent with that. Gara et al. also provided evidence from functional studies that *HABP2* is a tumor suppressor gene, and that the G534E variant results in loss of function. It remains possible that the G534E variant in *HABP2* is a susceptibility locus for cancer, but further studies are required to determine that. Other associations have been reported for the G534E variant of *HABP2* including thrombophilia, carotid stenosis and venous thromboembolism [29, 30], but neither the proband nor her son (who also carries this variant) have hematological or vascular disorders.

We also examined whole exome data for other missense mutations in *HABP2* and for variants which are thought to play a role in FNMTC in the known susceptibility genes, *SRGAP1*, *SRRM2*, *FOXE1* and *NKX2.1*. However, none of those genes had pathogenic variants identified in our study. Thus, the genetic basis of FNMTC in these kindreds remains to be determined.

Strengths of our study include the number of FNMTC kindreds recruited, and the use of both whole exome and Sanger sequencing to identify pathogenic mutations. A limitation is that not all members of all kindreds have undergone exome sequencing to date. Many of the kindreds studied had only two affected members which, as noted above, could occur by chance.

## Conclusions

In conclusion, we report that the G534E variant in *HABP2* is uncommon in FNMTC kindreds. Its frequency in general population indicates that it is a common polymorphism, and its role (if any) in the pathogenesis of thyroid cancer remains to be determined. Therefore the clinical yield of sequencing this variant in affected kindreds is likely to be small. The genetic basis of nonsyndromic FNMTC remains largely unknown, and more research is urgently required to elucidate this. In the meantime, the management of nonsyndromic FNMTC kindreds remains primarily clinical, as genetic testing has a minimal contribution to offer.

## Abbreviations

FNMTC, familial nonmedullary thyroid cancer; FOXE1, Forkhead Box E1; GWA, Genome Wide Association; HABP2, hyaluronan binding protein 2; MBq, megabecquerels; MTC, medullary thyroid cancer; NKX2-1, NK2 Homeobox 1; NMTC, nonmedullary thyroid cancer; PTC, papillary thyroid cancer; SRGAP1, SLIT-ROBO Rho GTPase Activating Protein 1; SRRM2, Serine/Arginine Repetitive Matrix 2; TCGA, The Cancer Genome Atlas
